# Meconium microbiome associates with the development of neonatal jaundice

**DOI:** 10.1038/s41424-018-0048-x

**Published:** 2018-09-20

**Authors:** Tianyu Dong, Ting Chen, Richard Allen White, Xu Wang, Weiyue Hu, Yali Liang, Yuqing Zhang, Chuncheng Lu, Minjian Chen, Heidi Aase, Yankai Xia

**Affiliations:** 1 State Key Laboratory of Reproductive Medicine, Institute of Toxicology, School of Public Health, Nanjing Medical University, Nanjing, 211166 China; 2 Key Laboratory of Modern Toxicology of Ministry of Education, School of Public Health, Nanjing Medical University, Nanjing, 211166 China; 3 Nanjing Maternity and Child Health Care Institute, The Affiliated Obstetrics and Gynaecology Hospital with Nanjing Medical University, Nanjing Maternity and Child Health Hospital, Nanjing, 210004 China; 4 Fundamental and Computational Sciences Directorate, Pacific Northwest National Laboratory, Richland, Washington USA; 5 School of Public Health, Wannan Medical College, Wuhu, 241002 China; 6 Department of Child Development, Norwegian Institute of Public Health, 0403 Oslo, Norway

## Abstract

**Objective:**

Neonatal jaundice is a common disease that affects up to 60% of newborns. Gut microbiota mediated the excretion of bilirubin from the human body. However, the relationship between early gut microbiome and development of neonatal jaundice is not fully understood. Here we sought to characterize meconium microbiome of newborns and to clarify its association with risk of neonatal jaundice.

**Methods:**

We conducted a nested case–control study with 301 newborns providing meconium samples from 2014 to 2015. The main outcome was the development of neonatal jaundice at 42 day follow-up. 16S rRNA gene sequencing was performed to profile the meconium microbiome. LEfSe was employed to identify different features between control and case groups. Logistic regression was used to estimate the risk effect of early gut microbiome on neonatal jaundice.

**Results:**

Logistic regression models suggested that higher ɑ-diversity was significantly associated with lower risk of jaundice in cesarean infants (OR 0.72, 95% CI 0.52–0.98), but not in infants born naturally. Higher relative abundance of *Bifidobacterium pseudolongum* in newborn meconium was significantly associated with lower risk of jaundice both in cesarean-born infants and in the total subjects (OR 0.24, 95% CI 0.07–0.68; OR 0.55, 95% CI 0.31–0.95, respectively). Spearman’s correlations showed that relative abundance of *B. pseudolongum* was significantly correlated with ɑ-diversity (*P* < 0.01).

**Conclusion:**

Preventive and treatment methods implying early gut microbiome intervention could be promising for the management of neonatal jaundice.

## Introduction

Neonatal jaundice is a common disease that affects up to 60% of newborns^1^. It is defined as the condition of high total serum bilirubin levels and usually developed in the first week of life. Most of the instances of neonatal jaundice are considered physiologic without underlying disease^2^, which can be distinguished from pathologic jaundice. Physiologic jaundice does not cause serious problems in newborns, but studies have linked it to adverse health outcomes in later life, such as childhood asthma, type 1 diabetes, and impaired visual function^3–5^. A large population-based longitudinal study in Denmark highlighted the risk for infants with neonatal jaundice (excluding pathologic jaundice resulting from hemolytic diseases) to develop autism and other psychological developmental disorders^6^, which raises public concerns about the long-term health effects caused by physiologic jaundice.

The pathogenesis for neonatal physiologic jaundice can be attributed to the nature for newborns to produce more bilirubin and their limited ability to excrete it^2^. In the biological process of bilirubin metabolism, gut bacteria has an important role in mediating the transformation of conjugated bilirubin to unconjugated bilirubin^7^, and then unconjugated bilirubin is further turned into bilinogen to be excreted out of the body. Previous studies have documented the association between early gut microbiome and child health^8–10^. Although it is recognized that gut bacteria is essential for bilirubin metabolism, the role of meconium microbiome on the development of physiologic jaundice has not been fully clarified. In addition, other known risk factors for neonatal jaundice, including cesarean section and breastfeeding, can influence the shaping of early gut microbiome^11–13^. Taking these factors into consideration would help us better understand the relationship between gut microbiome and jaundice.

Here we conducted a nested case–control study with a total of 301 newborns to illuminate the association between meconium microbiome and subsequent diagnosis of neonatal jaundice. Stratification analysis based on delivery mode and feeding pattern was also included in the study. The aim of our study was to clarify the role of early gut microbiome in the development of neonatal jaundice, thereby providing a knowledge base for potential preventive and treatment measures relevant to early gut microbiome interventions.

## Methods

### Experimental Design

This work was approved by the Ethical Committee of Nanjing Medical University before the study. The study population was drawn from the NMU Mother and Child Cohort Study (Phase II), a prospective birth cohort designed to study prenatal risk factors on pregnancy and child health outcomes. Participants in the present study were mother–infant pairs recruited in early pregnancy from affiliated hospitals with Nanjing Medical University between February 2014 and November 2015. The exclusion criteria included the following: 1. Age < 20 or > 45 years old; 2. Unwilling to register or deliver in these core hospitals; 3. No intention of long-term residence in the research area during pregnancy. All participants were regularly followed up at each trimester and at 42 days after delivery. Signed informed consent was obtained from the mothers at enrollment. The whole study was conducted in accordance with the Declaration of Helsinki of the World Medical Association.

Of the 542 pregnant women invited to the present study, 515 (95.02%) agreed to participate. During the follow-up, 72 pregnant women were lost, giving a follow-up rate of 86.02%. In the present study, 142 mother–infant pairs were removed from further analysis; of these, 12 resulted from assisted reproductive technology, 8 children had birth defects, 18 had neonatal diseases other than jaundice during the first 42 days after birth, 57 did not provide meconium samples, 11 had incomplete clinical or questionnaire data, and 36 newborns had pathologic jaundice. Pathologic jaundice is mainly caused by underlying diseases such as hemolytic diseases and liver diseases, which were not the focus of this study. Using a nested case–control design, eligible cases were identified and then matched with healthy controls by centers. Finally, a total of 301 mother–infant pairs remained in the present study.

### Outcome Measurements and Covariate Data Collection

The definition of neonatal jaundice was based on parental answers to the question “Has your child ever been diagnosed with neonatal jaundice by a doctor” in the questionnaire finished at the 42 days’ interview. If the answer was ‘yes,’ parents were asked to provide detailed information about time of diagnosis, duration, types of jaundice, medication, and hospitalization, in order to confirm physiologic jaundice. Information about feeding pattern and infant use of antibiotics were also obtained at that time. Clinical data from pregnancy and delivery were obtained from the hospital database. Premature rupture of membranes and gestational diabetes mellitus were reported to affect maternal and fetal gut microbiome^14,15^. Subclinical hypothyroidism in pregnancy was commonly found and involved autoimmunity^16,17^, which might influence gut microbiota (Table 1). Thus, these three factors were taken into consideration. Other covariates included maternal characteristics recorded in questionnaires at each interview: smoking and alcohol use during pregnancy, antibiotics use during pregnancy, reproductive history, and child feeding pattern.

**Table 1:** Characteristics of the study population

Variables^a^	Controls	Cases	*P*-value
	(*n* = 160)	(*n* = 141)	
Maternal age (year)	29.37 ± 3.91	29.48 ± 4.02	0.80
Center			
Nanjing	128 (80.0)	109 (77.3)	0.58
Suzhou	32 (20.0)	32 (22.7)	
Antibiotics to mother	4 (2.5)	7 (5.0)	0.36
Maternal smoking	8 (5.0)	2 (1.4)	0.11
Maternal alcohol consumption	7 (4.4)	6 (4.3)	1.00
Multipara	41 (25.6)	38 (27.0)	0.80
Premature rupture of membranes	35 (21.9)	39 (27.7)	0.28
Gestational diabetes mellitus	33 (20.6)	29 (20.6)	1.00
Subclinical hypothyroidism in pregnancy	34 (21.3)	21 (14.1)	0.18
Gestational age (day)	277.46 ± 6.46	274.79 ± 6.99	**<** 0.001***
Gender			
Male	80 (50.0)	74 (52.5)	0.73
Female	80 (50.0)	67 (47.5)	
Delivery mode			
Vaginal	103 (64.4)	100 (70.9)	0.27
Cesarean	57 (35.6)	41 (29.1)	
Birth weight (g)	3488.63 ± 381.64	3351.99 ± 341.90	**<** 0.01**
Apgar score			
1 min	9.97 ± 0.21	9.90 ± 0.56	0.16
5 min	9.99 ± 0.08	9.99 ± 0.17	0.59
Feeding pattern			
Breast milk	87 (54.4)	90 (63.8)	0.02*
Mixed	63 (39.4)	50 (35.5)	
Formula	10 (6.2)	1 (0.7)	
Antibiotics to newborn	1 (0.6)	3 (2.1)	0.34

^a^Data was presented as mean ± SD or *n* (%).

Boldface indicates statistical significance (*p < 0.05, **p < 0.01, ***p < 0.001).

### Sample Collection and DNA Extraction

Meconium samples were all first feces samples, which were collected by well-trained nurses from diapers into sterile tubes within 12 h after birth. These samples were immediately stored at − 80 °C until DNA extraction. Total DNA was extracted from meconium samples using the QIAamp Fast DNA Stool Mini Kit according to manufacturer’s instructions (Qiagen, Germany). All experiments were carried out on a sterile bench. DNA concentration and purity was determined by a NanoDrop 2000 (Thermo Fisher Scientific, Wilmington, DE).

### 16S rRNA Gene Sequencing and Quality Control

Hypervariable region V3 of the 16 S rRNA gene was amplified and then sequenced by HiSeq2500 PE250 platform in Novogene Bioinformatics Technology Co. Ltd (Beijing, China). After removing barcode and primer sequences, raw reads were merged using FLASH software (V1.2.7, http://ccb.jhu.edu/software/FLASH/). QIIME 1.9.1 64 bit was used to obtain quality-filtered reads and to cluster them into operational taxonomic units (OTUs) with Greengenes OTUs (16 S) v13_8 as the reference^18^. Finally, a total of 17,997,184 (mean 59,791.309 ± 9581.170; Min 29,699, Max 85,436) sequences remained and each sample was normalized to 29699 sequences for further analysis.

### Microbiome Analysis

Relative abundance of each bacterial level from phylum to genus was calculated in QIIME 1.9.1 64 bit. Shannon index was calculated to assess ɑ-diversity within samples. β-Diversity was visualized by PCoA (principal coordinates analysis) plot based on unweighted and weighted unifrac distance matrix. LEfSe (linear discriminant analysis effect size) was used to discover the features contributing to the most variation between control and case groups (LDA (linear discriminant analysis) > 2.5). Predicted metagenome of the 16S rRNA OTU data was obtained by PICRUSt and then was categorized by function according to KEGG Orthology.

### Statistical Analysis

All statistical analyses were carried out using R v3.4.0. For clinical data and characteristics, comparison of continuous univariate variables and ratios were performed by Student’s *t*-test, Fisher’s exact test, and *χ*^2^-test, respectively. For microbiome data, difference of Shannon index and relative abundance of *Bifidobacterium pseudolongum* was examined by Mann–Whitney test. Relative proportions of functional genes between control and case groups were compared by Welch’s *t*-test with corrected *P*-value in STAMP 2.1.3. Logistic regression models were employed for multivariable analysis and calculating odds ratio (OR) with corresponding 95% confidence interval (CI) adjusting for potential confounders. As the relative abundance of defined features by LEfSe were low (< 0.001) and outliers existed, these continuous variables were transformed into binary variables (low abundance and high abundance) based on the median of the total samples before including in the logistic regression models. Heterogeneity test was performed by “meta” R package. Spearman’s correlation analysis between relative abundance and Shannon index was conducted using cor.test function in R software. A *P*-value < 0.05 was considered significant.

## Results

### Characteristics of Study Population

A total of 141 eligible infants with physiologic jaundice were identified in the cohort. The eligible infants were matched with 160 controls without diagnosis of jaundice or any other neonatal diseases in their first month of life. The incidence of physiologic jaundice in the sample was 46.84%. Demographic characteristics of the infants and their mothers are shown in Table 1. Among all participants, 11 mothers used antibiotics during pregnancy and 4 infants used antibiotics after birth. Through univariate analysis, we found that gestational age and birth weight of the cases were significantly lower than those of the controls (*P* < 0.001 and *P* < 0.01, respectively). Feeding patterns also varied between the two groups (*P* = 0.02). No significant differences were observed in infant gender, maternal age at delivery, delivery mode, smoking status, or alcohol consumption between the groups.

### Variability and Diversity of the Meconium Microbiome

**Fig. 1:**
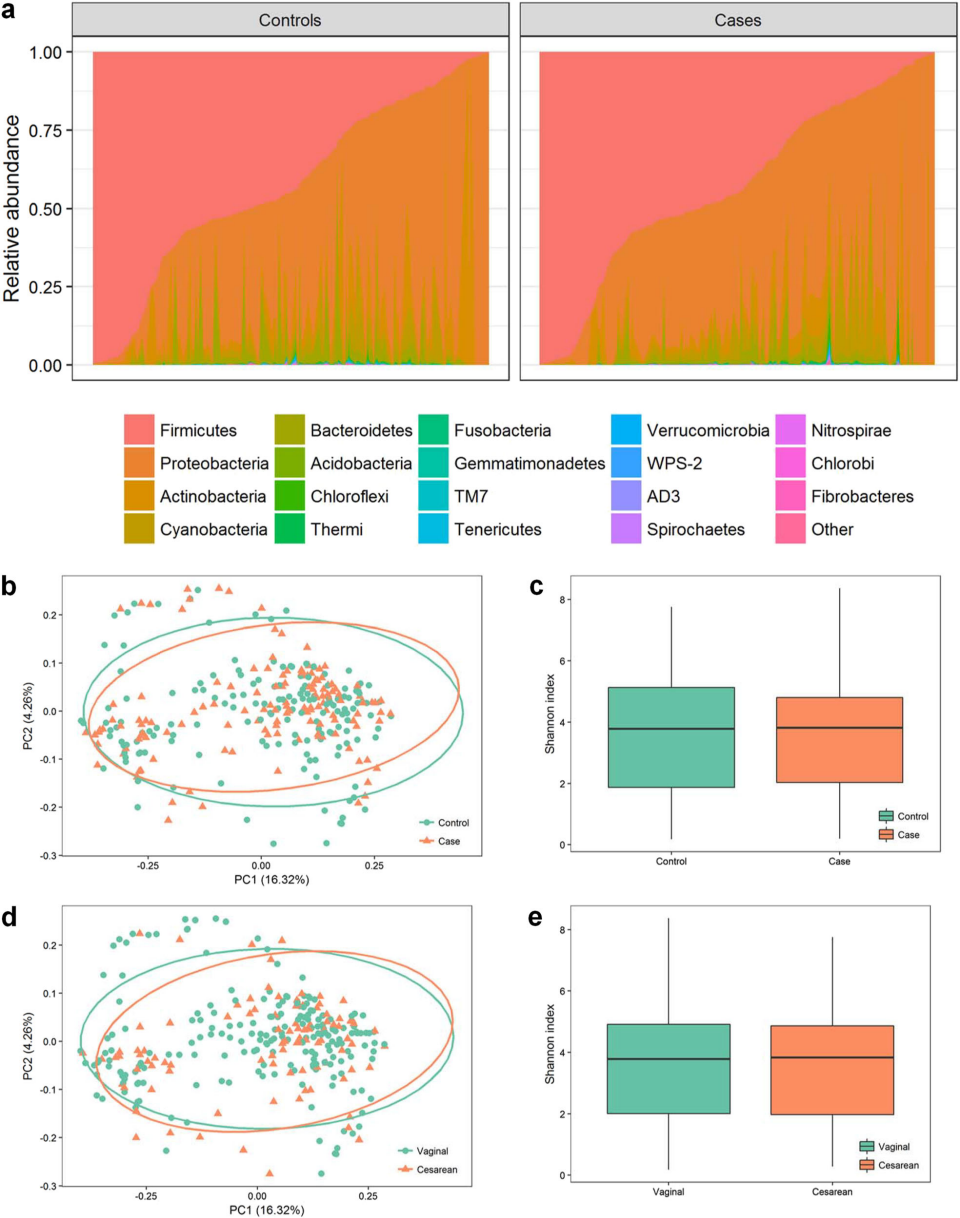
Variability and diversity of the meconium microbiome. **a** Constitution of microbiota among individuals sorted by relative abundance of Firmicutes. **b** PCoA plot based on unweighted unifrac distance by controls and cases. **c** Boxplot of Shannon index by controls and cases. **d** PCoA plot based on unweighted unifrac distance by delivery mode. **e** Boxplot of Shannon index by delivery mode

The meconium microbiome was dominated by *Firmicutes* and *Proteobacteria* (Fig. 1a). Large variation in the microbiome constitution among individuals could be observed (Fig. 1a). β-Diversity of all the samples divided by case and control group was visualized in PCoA plot based on unweighted unifrac distance. However, samples from the two groups were not separated observably **(**Fig. 1b). Similar results were found in PCoA plot based on weighted unifrac distance (Supplementary Fig. 1a, 1b). We also compared ɑ-diversity using Shannon index and the difference was not significant (*P* = 0.64) (Fig. 1c). As delivery mode was acknowledged as an important factor associated with newborn microbiome, we divided the samples into vaginal and cesarean groups. No significant differences in ɑ-diversity or β-diversity were observed between the two groups either (*P* = 0.95) (Fig. 1d, e).

### Alpha Diversity of Early Gut Microbiome and Risk of Neonatal Jaundice

A logistic regression model was employed to assess the impact of ɑ-diversity of early gut microbiome on the development of physiologic jaundice. Shannon index was included in the model to represent ɑ-diversity of meconium microbiome. Higher birth weight, longer gestational age and formula feeding (compared with breastfeeding) were all associated with lower risk of physiologic jaundice after adjusting for covariates (OR 0.9992, 95% CI 0.9985–0.9999; OR 0.94, 95% CI 0.90–0.98; and OR 0.094, 95% CI 0.005–0.559, respectively) (Table 2). However, Shannon index was not associated with the outcome (OR 0.94, 95% CI 0.83–1.07) (Table 2).

**Table 2:** Association of selected variables and risk of physiologic jaundice

Variables	OR^a^	95% CI	*P*-value
Maternal age (year)	1.00	0.93–1.09	0.91
Gestational age (day)	0.94	0.90–0.98	<0.01**
Antibiotics to mother	2.01	0.53–8.61	0.32
Premature rupture of membranes	1.27	0.70–2.33	0.43
Gestational diabetes mellitus	0.97	0.51–1.82	0.92
Pregnancy combined with hypothyroidism	0.71	0.36–1.38	0.31
Delivery mode			
Vaginal	1.00	Ref.	Ref.
Cesarean	0.66	0.37–1.17	0.16
Birth weight (g)	0.9992	0.9985–0.9999	0.04*
Feeding pattern			
Breast milk	1.00	Ref	Ref
Mixed	0.71	0.42–1.19	0.19
Formula	0.094	0.005–0.559	0.03*
Antibiotics to newborn	3.71	0.41–82.86	0.29
Shannon	0.94	0.83–1.07	0.36

^a^Adjusted for center, delivery mode, gender, feeding pattern, gestational age, birth weight, premature rupture of membranes, gestational diabetes mellitus, subclinical hypothyroidism in pregnancy, smoking, alcohol, maternal age, 1 min apgar score, 5 min apgar score, multipara, antibiotics to mother, and antibiotics to newborn

Boldface indicates statistical significance (*p < 0.05, **p < 0.01)

*CI* confidence interval, *OR* odds ratio

We further conducted stratification analysis by delivery mode to control its potential impacts on microbiome. The results of the logistic regression analysis suggested that higher Shannon indices were significantly associated with lower risk of physiologic jaundice after adjusting for potential confounders (OR 0.72, 95% CI 0.52–0.98) in infants delivered by cesarean section (Table 3). For vaginal delivered infants, no such association was observed (OR 0.98, 95% CI 0.83–1.15) (Table 3). Heterogeneity test indicated moderate heterogeneity between vaginal and cesarean subgroups (*τ*^2^ = 0.03, *H* = 1.68, *I*^2^ = 64.40%, *P* = 0.09) (Table 3).

**Table 3:** Stratification analysis on the association between Shannon index and risk of physiologic jaundice by delivery mode and feeding patterns

Variables	Shannon index			Heterogeneity	
	OR	95% CI	*P*-value	*I*^2^%	*P*-value
Delivery mode^a^					
Vaginal	0.98	0.83–1.15	0.80	64.40	0.09
Cesarean	0.72	0.52–0.98	0.04*		
Exclusive breastfeeding^b^					
Yes	0.98	0.83–1.17	0.86	36.70	0.21
No	0.81	0.63–1.03	0.10		

^a^Adjusted for center, gender, feeding pattern, gestational age, birth weight, premature rupture of membranes, gestational diabetes mellitus, subclinical hypothyroidism in pregnancy, smoking, alcohol, maternal age, 1 min apgar score, 5 min apgar score, multipara, antibiotics to mother, and antibiotics to newborn

^b^Adjusted for center, delivery mode, gender, gestational age, birth weight, premature rupture of membranes, gestational diabetes mellitus, subclinical hypothyroidism in pregnancy, smoking, alcohol, maternal age, 1 min apgar score, 5 min apgar score, multipara, antibiotics to mother and antibiotics to newborn

Boldface indicates statistical significance (*p < 0.05).

We also stratified the study population by feeding patterns. As the sample size of formula feeding infants was limited, we divided all subjects into exclusive breast-feeding and non-exclusive breast-feeding groups. Results showed that the Shannon index was not associated with jaundice whether the infant was exclusively breastfed or not (OR 0.98, 95% CI 0.83–1.17, OR 0.81, 95% CI 0.63–1.03, respectively) (Table 3).

### Differences of microbiome between controls and cases in cesarean infants

**Fig. 2:**
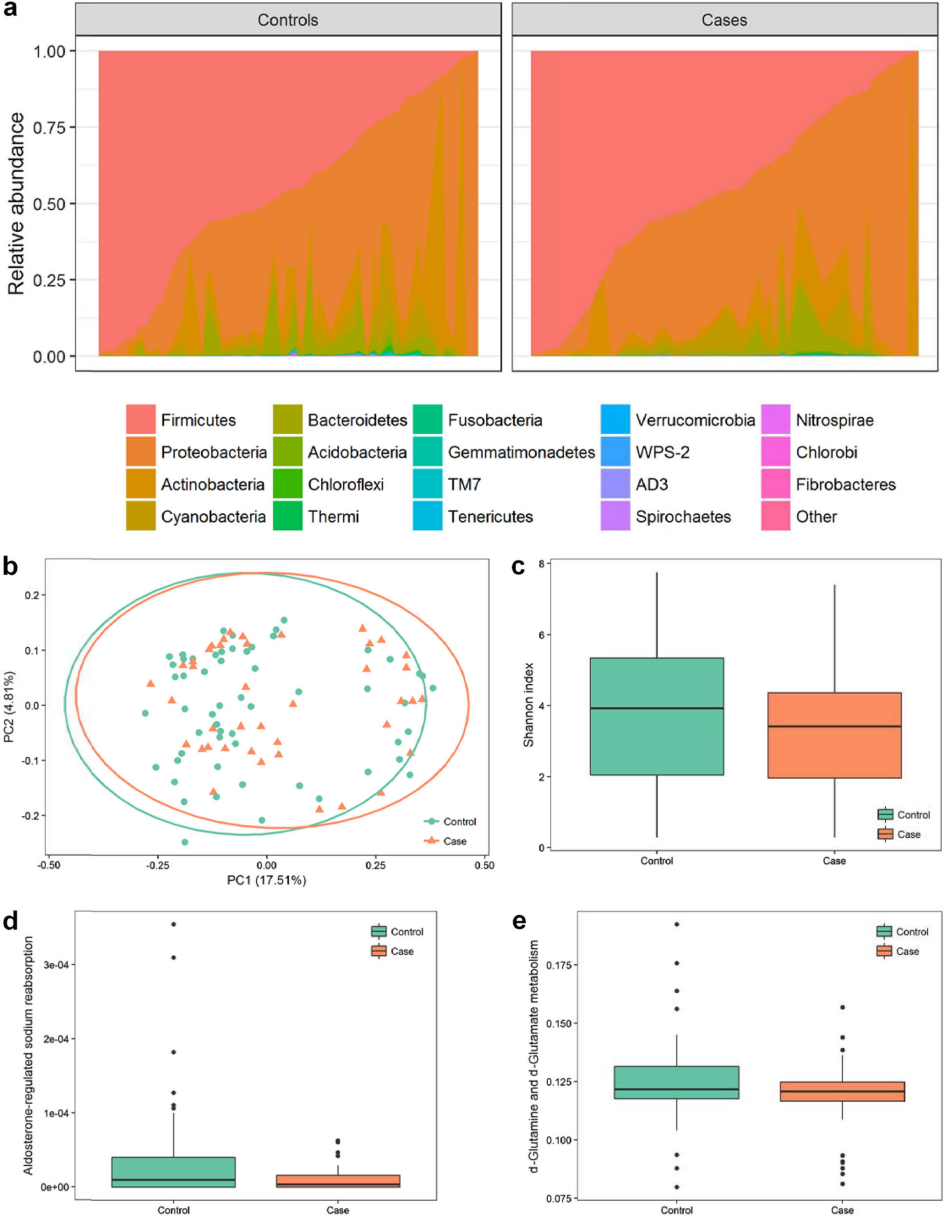
Variability and diversity of the meconium microbiome of cesarean infants. **a** Constitution of microbiota among cesarean newborns sorted by relative abundance of Firmicutes. **b** PCoA plot based on unweighted unifrac distance by controls and cases. **c** Boxplot of Shannon index by controls and cases. **d**, **e** Boxplot of proportion of functional genes by controls and cases

It was an interesting finding that ɑ-diversity of the microbiome might be a protective factor against jaundice only in cesarean-born infants. Thus, we further compared the microbiome between controls and cases in the cesarean group. No significant difference was observed in the incidence rates of the vaginal and the cesarean group (49.26% and 41.84%, respectively; *P* = 0.27). Stacking area map generated from proportion of bacteria at the phylum level showed similar results with that found in the total population (Fig. 2a). Large variations between individuals in abundancy of bacteria could be observed and differences might also exist for the less abundant bacteria (Fig. 2a). No marked separation was found in PCoA plot based on unweighted unifrac distance (Fig. 2b). Similar results were also found in PCoA plot based on weighted unifrac distance (Supplementary Fig. 1c).The Shannon indices were lower in the cases compared with controls, but the *P*-value was not significant (*P* = 0.20) (Fig. 2c). PICRUSt was used to predict metagenome of the 16S rRNA data followed by function categorizing. Compared with the control group, aldosterone-regulated sodium reabsorption and d-Glutamine and d-glutamate metabolism were both significantly decreased (corrected *P* < 0.01 and *P* = 0.035, respectively) (Fig. 2d, e).

**Fig. 3:**
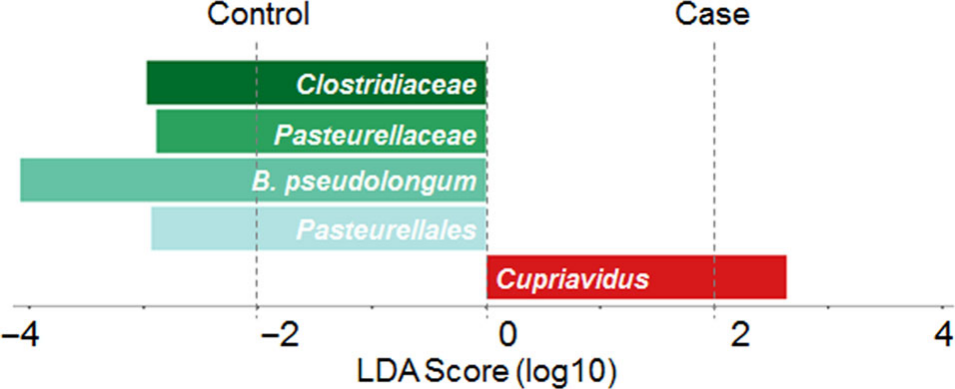
Bar plot of the LDA Score (log10) between control and case groups in cesarean infants. The left panel indicated higher abundance in the control group, and the right panel indicated higher abundance in the case group. *B. pseudolongum* is the abbreviation that stands for *B. pseudolongum*

Finally, we conducted LEfSe to discover distinctive features at all levels. According to the threshold LDA > 2.5, five features were found to be significantly different between controls and cases. Four features were more abundant in the control group (*Pasteurellales*, *B. pseudolongum*, *Pasteurellaceae*, and *Clostridiaceae*) and only one feature was more abundant in the case group (*Cupriavidus*) (Fig. 3).

### Relative abundance of *Bifidobacterium pseudolongum* and risk of neonatal Jaundice

Notably, *B. pseudolongum* exhibited the highest LDA score in LEfSe (LDA = 4.08) (Fig. 3). In order to assess the effects of the *B. pseudolongum* on the outcome in newborns from cesarean section, we categorized the relative abundance of *B. pseudolongum* into more abundant and less abundant groups based on the median in the total sample, and included it in the logistic regression model. The results showed a significantly lower risk of physiologic jaundice in the more abundant group compared with the less abundant group after adjusting for confounders (OR 0.24, 95% CI 0.07–0.68) (Table 4). As no differences in the relative abundance of *B. pseudolongum* were found between vaginal and cesarean groups (*P* = 0.21), we would also like to know whether this association only existed in cesarean-born infants or not. Therefore, we employed logistic regression models again in both vaginal born infants and the total sample. As shown in Table 4, more abundance of *B. pseudolongum* was significantly associated with the outcome in the total sample, while no association was found for vaginal born infants (OR 0.55, 95% CI 0.31–0.95, OR 0.68, 95% CI 0.34–1.34, respectively). Heterogeneity test suggested moderate heterogeneity between vaginal born and cesarean section subgroups (*τ*^2^ = 0.34, *H* = 1.60, *I*^2^ = 61.20%, *P* = 0.11) (Table 4).

**Table 4:** Association of relative abundance of *B. pseudolongum* and risk of physiologic jaundice

Relative abundance^a^	OR	95% CI	*P*-value
Pooled			
Low	1	Ref.	Ref.
High	0.55	0.31–0.95	0.03*
Vaginal			
Low	1	Ref.	Ref.
High	0.68	0.34–1.34	0.27
Cesarean			
Low	1	Ref	Ref
High	0.24	0.07–0.68	**<** 0.01**

^a^Adjusted for center, gender, feeding pattern, gestational age, birth weight, premature rupture of membranes, gestational diabetes mellitus, subclinical hypothyroidism in pregnancy, smoking, alcohol, maternal age, 1 min apgar score, 5 min apgar score, multipara, antibiotics to mother, and antibiotics to newborn

Boldface indicates statistical significance (*p < 0.05, **p < 0.01).

### Correlation between relative abundance of *B. pseudolongum* and ɑ-diversity

Spearman’s correlation analysis was used to explore the correlation between relative abundance of *B. pseudolongum* and ɑ-diversity. Results showed that relative abundance of *B. pseudolongum* was significantly correlated with ɑ-diversity (*P* < 0.01).

## Discussion

Despite high incidence of neonatal physiologic jaundice, newborns with the condition may not be treated properly due to a misunderstanding of the term “physiologic” as not serious, opposed to “pathologic”. Dennery et al.^2^ reviewed that bilirubin was cellular-toxic and neurotoxic. Many studies have highlighted the association between neonatal jaundice and psychological disorders, including autism spectrum disorder and attention deficit hyperactivity disorder^6,19^. Considering the potential health effects, more attention should be paid to control the incidence.

In this study, we used a nested case–control design, and enrolled a total of 301 participants with parental-reported diagnosis of physiologic jaundice. Results showed that higher birth weight, longer gestational age, and formula feeding were all associated with lower risk of physiologic jaundice after adjusting for covariates, which were consistent with previous report^20,21^. When stratification analysis by delivery mode was performed, we observed a significant association between higher Shannon index and lower risk of jaundice in cesarean-born infants. It has been reported that higher ɑ-diversity was associated with lower risk of atopic eczema, neonatal sepsis, and necrotizing enterocolitis^10,22,23^. Our study suggested that higher ɑ-diversity of gut microbiome could also be a protective factor for cesarean-born infants at risk of jaundice. This was an interesting finding as it was not found in vaginal born infants. Jakobsson et al.^24^ conducted a time-series study to describe the dynamics of gut microbiome diversity of infants by delivery mode in the first 2 years after birth. They found delayed colonization of microbiota in cesarean-born infants compared with vaginal born infants, although no significant difference in ɑ-diversity was observed in the first week. We speculate that for cesarean-born infants, delayed colonization also delays the duration of immature meconium microbiome, and those with lower diversity of meconium microbiome were put into higher risk for jaundice compared with those with high diversity of meconium microbiome. For vaginal born infants with relatively lower diversity of microbiome, rapid colonization of bacteria compensated deficiency of diversity, which diluted the effects of low diversity on the development of jaundice. This finding could also explain the reason why cesarean-born infants were at higher risk to develop jaundice compared with vaginal born infants in a previous study^13^.

LEfSe identified three features that were significantly different between controls and cases in all subjects (LDA > 2.5) (Supplementary Fig. 2). There was only one feature more abundant in the case group (*Clostridium perfringens*). Vítek et al.^21^ collected stool samples from 5-day-old newborns and isolated a strain of *C. perfringens* capable of reducing bilirubin to urobilinoids, which supported current findings. It was highly possible that the increased abundance of *C. perfringens* was the feedback to high bilirubin levels in jaundice infants. As *C. perfringens* is acknowledged as an etiologic agent and was harmful to human health, its role in the pathogenesis of neonatal jaundice should be clarified in future studies.

Then we focused on the microbiome of cesarean-born infants. Five features were discovered by LEfSe, and the relative abundance of *B. pseudolongum*, which exhibited the higherst LDA score, was significantly associated with jaundice. Similar results were found in the total population, but not in the vaginal born infants. In addition, correlation analysis showed that relative abundance of *B. pseudolongum* was significantly correlated with ɑ-diversity. *B. pseudolongum* belongs to the *Bifidobacterium*, and was first isolated from an anemic Kenyan infant^25^. *Bifidobacterium* strains were identified to exert antimicrobial activity against potential pathogenic microorganisms in infant gut^26,27^, which could benefit the colonization of healthy bacteria^28^. Despite the limited information about detailed function of *B. pseudolongum*, we speculate that it may act like other known *Bifidobacterium* strains by defending pathogens to promote the maturation of early gut microbiome and finally lower the risk of jaundice. However, due to the single time point of sample collection in this study, we could only confirm the correlation between *B. pseudolongum* and ɑ-diversity, but not causality. We should notice that clinicians are using probiotic supplementation including *Bifidobacterium* to prevent and treat neonatal jaundice around the world^29^. Two meta-analyses on randomized controlled trials of probiotics therapy for the management of neonatal jaundice conservatively concluded that probiotic supplementation could be effective in treating neonatal jaundice^29,30^. Large population-based longitudinal time-series studies are needed to confirm the causal association among *B. pseudolongum*, ɑ-diversity and neonatal jaundice.

The present study included some limitations. First, the outcome measures were based on parental reports, which could result in potential recall bias. Second, the sample size was relatively small. The effects caused by early microbiome in the total sample could be smoothed out. Third, we only had meconium samples at a single time point and could not confirm the causal association between *B. pseudolongum* and ɑ-diversity. Time-series sample collection design is expected in the future.

## Study Highlights

### What is current knowledge?

- ✓ Neonatal jaundice is a commonly found disease in newborns.
- ✓ Gut bacteria mediates the bilirubin metabolism.

### What is new here?

- ✓ Gut microbiome is associated with the development of neonatal jaundice especially in C-section babies.
- ✓ Higher relative abundance of *B. pseudolongum* is associated with lower risk of jaundice.

## Electronic supplementary material

Supplementary Figure 1

Supplementary Figure 2

Supplementary Figure legend
