# Supplementary figures and images for: Meconium microbiome associates with the development of neonatal jaundice
Source: Clin Transl Gastroenterol. 2018 Sep 20;9(9):182. doi: 10.1038/s41424-018-0048-x (PMC6147945; doi:10.1038/s41424-018-0048-x)

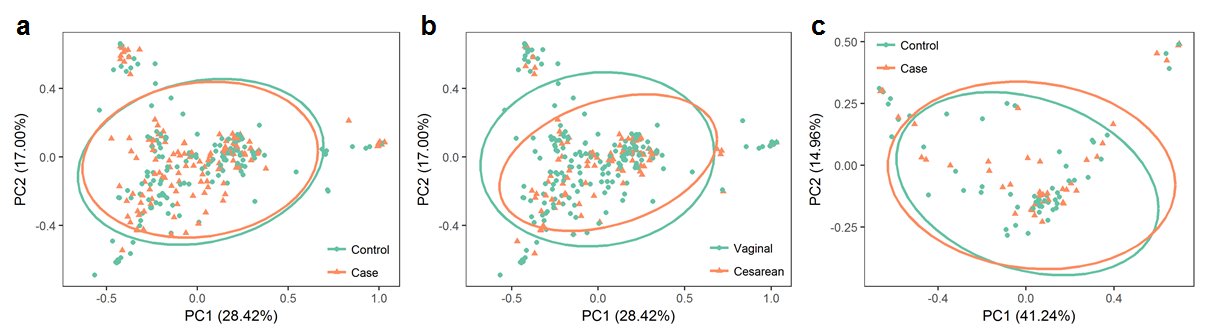

Supplement: Supplementary file 1 — Supplementary Figure 1 [file 41424_2018_48_MOESM1_ESM.tif]

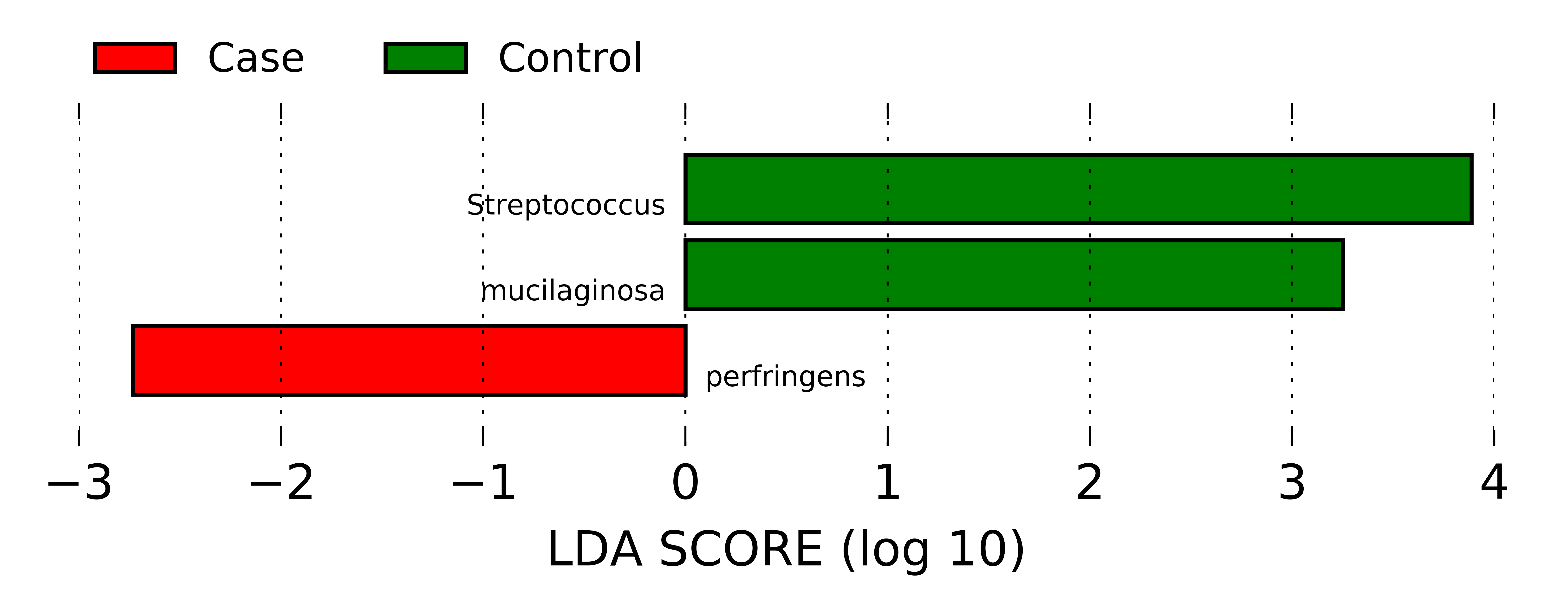

Supplement: Supplementary file 2 — Supplementary Figure 2 [file 41424_2018_48_MOESM2_ESM.png]
