# Supplementary material for: Meconium microbiome associates with the development of neonatal jaundice
Source: Clin Transl Gastroenterol. 2018 Sep 20;9(9):182. doi: 10.1038/s41424-018-0048-x (PMC6147945; doi:10.1038/s41424-018-0048-x)
Supplement: Supplementary file 3 — Supplementary Figure legend [file 41424_2018_48_MOESM3_ESM.docx]

**Supplementary Fig. 1:** Beta-diversity of meconium microbiome. (a) PCoA plot based on weighted unifrac distance by controls and cases in all infants; (b) PCoA plot based on weighted unifrac distance by delivery mode in all infants; (c) PCoA plot based on weighted unifrac distance by controls and cases in cesarean infants.

**Supplementary Fig. 2:** Bar plot of the LDA Score (log10) between control and case groups in all infants. The left panel indicated higher abundance in the control group, and the right panel indicated higher abundance in the case group.
